# Supplementary material for: Restoration of breathing after opioid overdose and spinal cord injury using temporal interference stimulation
Source: Commun Biol. 2021 Jan 25;4:107. doi: 10.1038/s42003-020-01604-x (PMC7835220; doi:10.1038/s42003-020-01604-x)
Supplement: Supplementary file 2 — Supplementary Information [file 42003_2020_1604_MOESM2_ESM.pdf]

Title: Restoration of breathing after opioid overdose and spinal cord injury using temporal interference stimulation

Authors: Michael D. Sunshine[1,2,3,4], Antonino M. Cassarà[5], Esra Neufeld[5], Nir Grossman[6,7], Thomas H. Mareci[8], Kevin J. Otto[9,10,11,12,13], Edward S. Boyden[14,15], *David D. Fuller[2,3,4]

Affiliations: [1]Rehabilitation Science PhD Program, University of Florida; Gainesville FL, USA. [2]Department of Physical Therapy, University of Florida; Gainesville FL, USA. [3]Breathing Research and Therapeutics Center, University of Florida; Gainesville FL, USA. [4]McKnight Brain Institute, University of Florida; Gainesville FL, USA. [5]Foundation for Research on Information Technologies in Society (IT'IS), Zurich, Switzerland. [6]Division of Brain Sciences, Imperial College London; London, United Kingdom. [7]United Kingdom Dementia Research Institute, Imperial College London; London, United Kingdom. [8]Department of Biochemistry and Molecular Biology, University of Florida; Gainesville FL, USA. [9]J. Crayton Pruitt Family Department of Biomedical Engineering, University of Florida; Gainesville FL, USA. [10]Department of Neuroscience, University of Florida; Gainesville FL, USA. [11]Department of Neurology, University of Florida; Gainesville FL, USA. [12]Department of Materials Science & Engineering, University of Florida; Gainesville FL, USA. [13]Department of Electrical & Computer Engineering, University of Florida; Gainesville FL, USA. [14]Departments of Brain and Cognitive Sciences, Media Arts and Sciences, and Biological Engineering, McGovern and Koch Institutes, MIT; Cambridge, MA USA. [15]Howard Hughes Medical Institute, Cambridge, MA USA.

* Corresponding author. David D. Fuller; email: dfuller@phhp.ufl.edu

---

SUPPLEMENTAL FIGURES

Supplementary Fig. 1: Localization of intramuscular wires used in the opioid-rescue experiment.

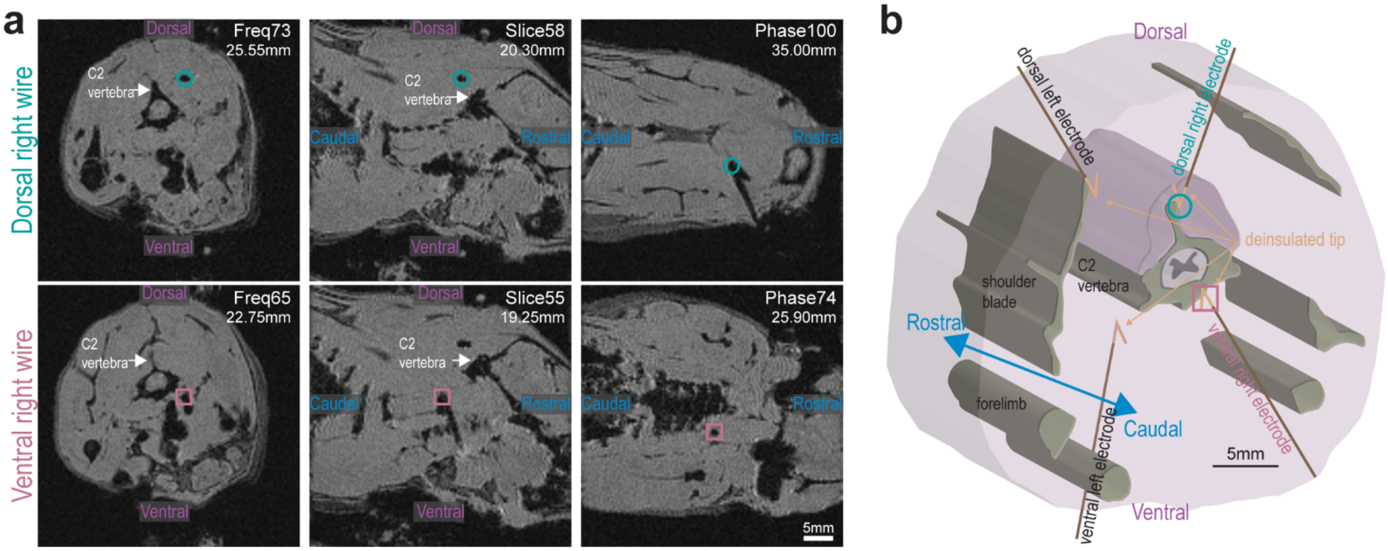

An ex vivo 4.7 tesla scan of the cervical cord and neck of 233 g male rat is shown in panel **a**. Data were collected using an isotropic gradient echo (30 ms repetition time, and 10 ms echo time) 44.8 x 44.8 x 44.8 mm^3 three-dimensional scan with 0.35 mm isotropic resolution. Coronal, axial, and sagittal slices show the dorsal (green circle) and ventral (pink square) tungsten wire electrodes on the right side of the animal. Panel **b** provides a 3D rendering, based on the MRI data, showing the location of the 4 wire locations in the muscles surrounding C2 and C3. The deinsulated end of the wires are indicated by the arrows.

Supplementary Fig. 2: Following opioid overdose and hypoventilation, TI stimulation maintains arterial blood oxygenation and prevents hypercapnia.

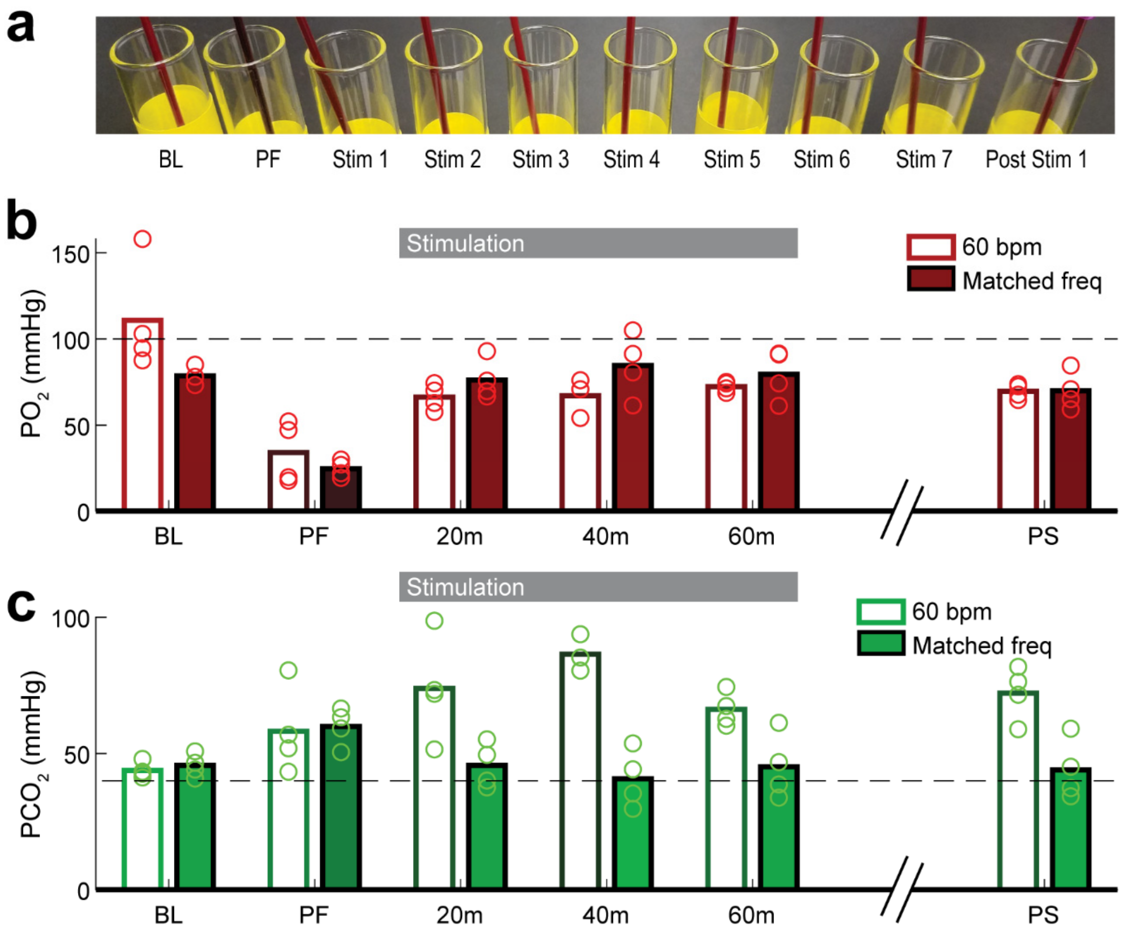

Two intramuscular TI stimulation methods were used. The first used waveforms at 5000 Hz and 5001 Hz to create a 1 Hz beat frequency, thereby activating the diaphragm at 60 breaths per minute. The second paradigm matched the TI-induced diaphragm bursting with the breathing rate that occurred spontaneously prior to the opioid overdose (average of 110±10 breaths per minute). This was accomplished using waveforms with a carrier frequency of 5000 Hz and an offset frequency determined based on the pre-opioid breathing rates (range: 1.6-1.933 Hz). Panel **a** shows a photograph of arterial blood samples taken over the course an entire experimental protocol. Following opioid overdose (PF or post-fentanyl) the arterial blood has a dark color indicating hypoxemia. Shortly after initiation of TI simulation (in this example using the “Matched freq” protocol) bright red coloration is restored, thereby providing a visual confirmation of blood oxygenation. Panel **b** shows that both forms of TI stimulation increase the partial pressure of arterial oxygen towards baseline. Panel **c** shows that TI stimulation producing a respiratory rate of 60 breaths per minute was not sufficient restore the partial pressure of arterial carbon dioxide (i.e., some degree of hypoventilation persisted). In contrast, TI stimulation producing diaphragm contraction at a rate of 110±10 breaths per minute prevented arterial hypercapnia.

Supplementary Fig. 3: Simulation of current steering behavior using epidural electrodes in 1.5 mm and 3 mm configurations.

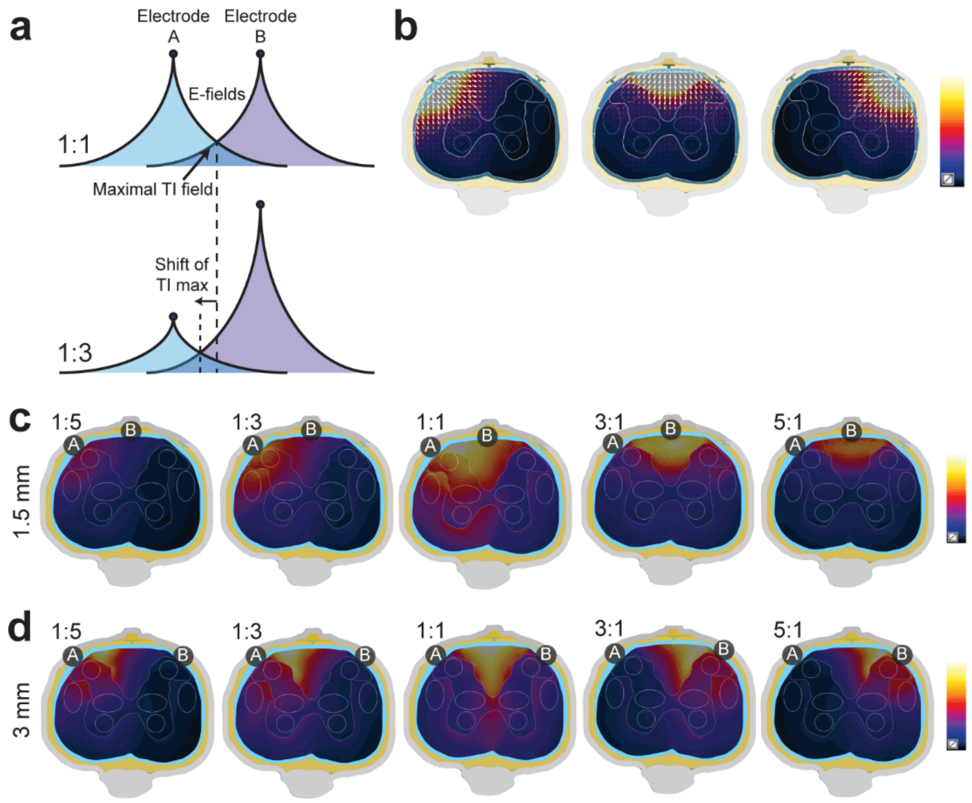

a The observed steering behavior (i.e., a shift of the stimulation site towards the electrode with the reduced current) supports the hypothesis that the amplitude of the TI modulation envelope (which is determined by the smaller of the two TI fields) correlates with the neural response. **b** Illustration of the electric field distributions for the two channels. **c-d** Illustration of the TI field steering as a function of the current ratio for the **c** '1.5 mm' and **d** '3 mm' configurations shown in Fig. 4 and 6.

Supplementary Fig. 4: Verification that neural substrate is required for diaphragm EMG activation during epidural TI stimulation.

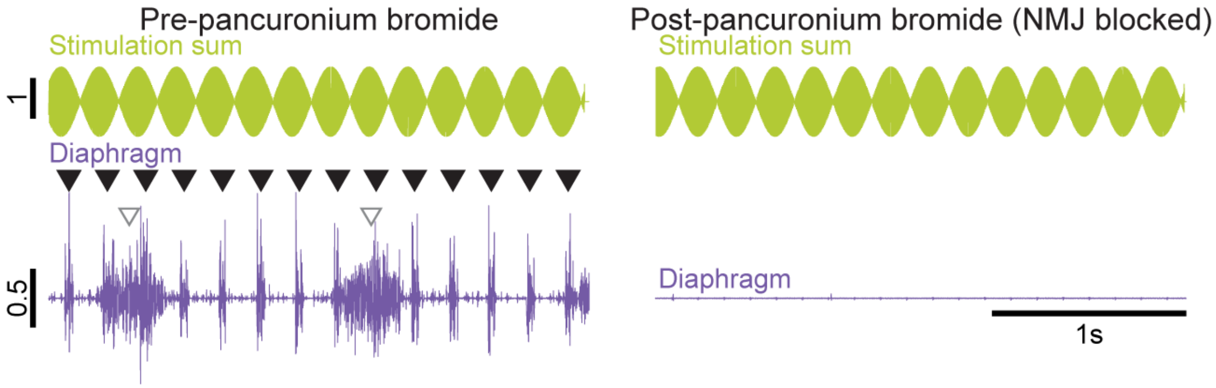

The top traces show the TI stimulation sums. In this example, TI was delivered using 5000 and 5005 Hz waveforms, thereby activating the diaphragm at 5 Hz. The bottom traces show diaphragm EMG activity during epidural TI stimulation before and after intravenous delivery of a pharmacologic agent (pancuronium bromide, 2.5 mg/kg) which blocks transmission at the neuromuscular junction (NMJ). At baseline (left panel), endogenous diaphragm bursting (white arrow heads) and TI-evoked diaphragm bursting (black arrowheads) can be observed. Both are completely abolished following pancuronium bromide (right panel). Scale bar units: stimulation (mA), diaphragm (mV).

Supplementary Fig. 5: Example EMG responses to epidural TI and square wave stimulation.

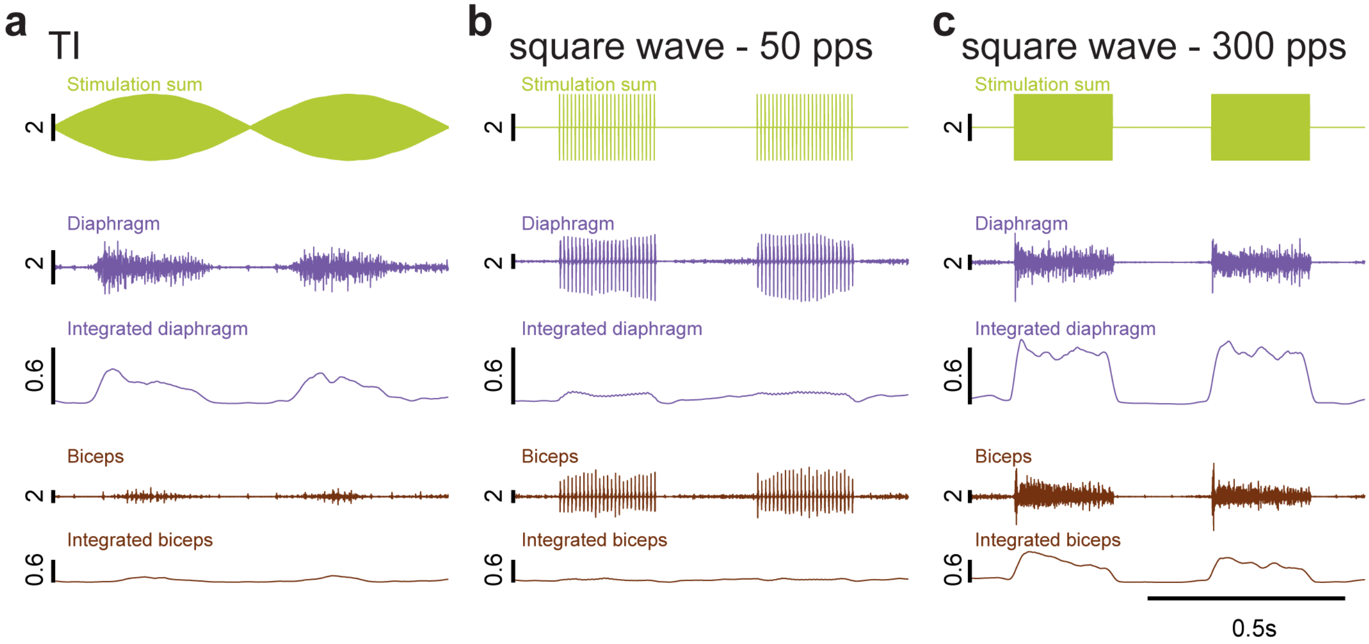

Panel **a** depicts epidural TI stimulation with frequencies of 5000 Hz + 5001 Hz. This produced a large diaphragm EMG burst in phase with the TI-offset, and a small activation of the biceps. The integrated diaphragm trace shows a ramping of activation that is similar to what occurs during spontaneous diaphragm activity, Panel **b** shows diaphragm and biceps activation during epidural square wave stimulation (50 pulse per second (pps), delivered in repeated 0.5 s trains. This produced brief evoked compound muscle action potentials in both muscles. Lastly, panel **c** shows epidural square wave stimulation delivered at 300 pps. This produced large unmodulated (i.e. fixed amplitude) bursts in the diaphragm as well as the biceps. Scale bar units are: stimulation (mA), diaphragm and biceps (mV), integrated diaphragm and biceps (arbitrary units).
