# Supplementary material for: Restoration of breathing after opioid overdose and spinal cord injury using temporal interference stimulation
Source: Commun Biol. 2021 Jan 25;4:107. doi: 10.1038/s42003-020-01604-x (PMC7835220; doi:10.1038/s42003-020-01604-x)
Supplement: Supplementary file 3 — Description of Additional Supplementary Files [file 42003_2020_1604_MOESM3_ESM.pdf]

Description of Additional Supplementary Files

Name: Supplementary Data 1

Description: This file contains spreadsheets that contain the original data used to create the figures in our manuscript. The file has been annotated so that users can understand the organization and recreate our analyses, as needed.

Name: Supplementary Movie 1

Description: Intramuscular TI stimulation restores ventilation following opioid induced apnea. In a spontaneously breathing urethane anesthetized rat intravenous fentanyl (30 mcg/kg) causes lasting apnea and reduction in peripheral oxygenation (SPO2). TI rescue stimulation produces a robust activation of the diaphragm and airflow, which restores SPO2 levels and attenuates drop in arterial blood pressure (ABP).

Name: Supplementary Movie 2

Description: Intramuscular TI stimulation robustly activated diaphragm while high and low frequency control stimulation do not effectively activate the diaphragm. Intramuscular TI stimulation in a urethane anesthetized ventilated rat overdosed on fentanyl (30 mcg/kg) robustly activates both hemi-diaphragms, while subsequent high frequency and low frequency stimulation at the same currents fail to cause phasic diaphragm contraction.
