# Supplementary material for: Restoration of breathing after opioid overdose and spinal cord injury using temporal interference stimulation
Source: Commun Biol. 2021 Jan 25;4:107. doi: 10.1038/s42003-020-01604-x (PMC7835220; doi:10.1038/s42003-020-01604-x)
Supplement: Supplementary file 7 — Reporting Summary [file 42003_2020_1604_MOESM7_ESM.pdf]

nature research

Corresponding author(s): David Fuller
Last updated by author(s): Oct 9, 2020

Reporting Summary

Nature Research wishes to improve the reproducibility of the work that we publish. This form provides structure for consistency and transparency in reporting. For further information on Nature Research policies, see our Editorial Policies and the Editorial Policy Checklist.

Statistics

For all statistical analyses, confirm that the following items are present in the figure legend, table legend, main text, or Methods section.

n/a	Confirmed	
[ ]	[x]	The exact sample size (n) for each experimental group/condition, given as a discrete number and unit of measurement
[ ]	[x]	A statement on whether measurements were taken from distinct samples or whether the same sample was measured repeatedly
[ ]	[x]	The statistical test(s) used AND whether they are one- or two-sided<br>*Only common tests should be described solely by name; describe more complex techniques in the Methods section.*
[x]	[ ]	A description of all covariates tested
[ ]	[x]	A description of any assumptions or corrections, such as tests of normality and adjustment for multiple comparisons
[ ]	[x]	A full description of the statistical parameters including central tendency (e.g. means) or other basic estimates (e.g. regression coefficient) AND variation (e.g. standard deviation) or associated estimates of uncertainty (e.g. confidence intervals)
[ ]	[x]	For null hypothesis testing, the test statistic (e.g. F, t, r) with confidence intervals, effect sizes, degrees of freedom and P value noted<br>*Give P values as exact values whenever suitable.*
[x]	[ ]	For Bayesian analysis, information on the choice of priors and Markov chain Monte Carlo settings
[x]	[ ]	For hierarchical and complex designs, identification of the appropriate level for tests and full reporting of outcomes
[x]	[ ]	Estimates of effect sizes (e.g. Cohen's d, Pearson's r), indicating how they were calculated

Our web collection on statistics for biologists contains articles on many of the points above.

Software and code

Policy information about availability of computer code

Data collection	In vivo data was collected using Spike2 version 8 (cambridge Electronic Design) using standard configuration file (i.e. no script).
Data analysis	In vivo data was analyzed with MATLAB 2019a. Scripts were written specifically to analyze data in this manuscript by M. Sunshine.

For manuscripts utilizing custom algorithms or software that are central to the research but not yet described in published literature, software must be made available to editors and reviewers. We strongly encourage code deposition in a community repository (e.g. GitHub). See the Nature Research guidelines for submitting code & software for further information.

Data

Policy information about availability of data

All manuscripts must include a data availability statement. This statement should provide the following information, where applicable:
- Accession codes, unique identifiers, or web links for publicly available datasets
- A list of figures that have associated raw data
- A description of any restrictions on data availability

The datasets generated during and/or analysed during the current study are available from the corresponding author on reasonable request.

Field-specific reporting

Life sciences study design

All studies must disclose on these points even when the disclosure is negative.

Sample size	The goal these experiments was to determine if a novel method of central nervous system stimulation (temportal interference) could effectively activate spinal respiratory motor neurons to restore ventilation after opioid overdose or spinal cord injury. Preliminary testing indicated that temporal interference could induce robust diaphragm muscle activation. We assumed an expected difference of means (e.g., stimulation vs. no stimulation) of 75%, expected standard deviation of 25%, power of 0.9 and alpha of 0.05. This enabled a power calculation suggesting that n=4 per group would be sufficient to statistically differentiate the impact of temporal interference for the primary outcome of diaphragm EMG activity.
Data exclusions	Experiments were excluded from analysis if it was determined that there was a mechanical failure (e.g., broken electrode array, stimulator battery failure, heating pad failure). We did not test for outliers in our data, and included all data from each set of experiments.
Replication	The primary finding of temporal interference - induced activation of the diaphragm muscle to sustain breathing was replicated as follows. We delivered the temporal interference stimulation using two fundamentally different electrode configurations (intramuscular or epidural), and both methods were shown to activate the diaphragm. Further, within the epidural stimulation group, the data were further replicated by testing different electrode configurations. We were able to reproducibility activate the left vs. right diaphragm based on variations in the electrode placement and/or current.
Randomization	Animals were studied in a randomized block design, whereby an animal from each group was studied in a random order (e.g . 1) intact, acute SCI, chronic SCI, then 2) acute SCI, intact, chronic SCI etc.).
Blinding	The primary experimentor was not blinded during data collection as the experimental conditions (e.g. spinal cord injury, muscle activation from stimulation) are overt. However, the experimenter was blinded during the data was analysis as follows. All data were evaluated using MATLAB 2019a, which provided a standardized tool to apply across all data. Once the MATLAB coding was established, all data from each study were "batch processed" identically to produce an unbiased result.

Reporting for specific materials, systems and methods

We require information from authors about some types of materials, experimental systems and methods used in many studies. Here, indicate whether each material, system or method listed is relevant to your study. If you are not sure if a list item applies to your research, read the appropriate section before selecting a response.

Materials & experimental systems

n/a	Involved in the study	
☒	☐	Antibodies
☒	☐	Eukaryotic cell lines
☒	☐	Palaeontology and archaeology
☐	☒	Animals and other organisms
☒	☐	Human research participants
☒	☐	Clinical data
☒	☐	Dual use research of concern

Methods

n/a	Involved in the study	
☒	☐	ChIP-seq
☒	☐	Flow cytometry
☒	☐	MRI-based neuroimaging

Animals and other organisms

Policy information about studies involving animals; ARRIVE guidelines recommended for reporting animal research

Laboratory animals	The animals in this study were Sprague-Dawley rats, roughly equal numbers of each sex were used in each experiment. Animals were obtained to control for body weight rather as the size of the animals could effect experimental outcomes.
Wild animals	The study did not involve wild animals
Field-collected samples	The study did not include samples collected in the field
Ethics oversight	The University of Florida Institutional Animal Care and Use Committee approved the work contained within this manuscript.

Note that full information on the approval of the study protocol must also be provided in the manuscript.
